# The Effect of Vitamin D on Metabolic Bone Disease and Chronic Diseases

**DOI:** 10.3390/nu15224775

**Published:** 2023-11-14

**Authors:** Salvatore Minisola, Daniela Merlotti

**Affiliations:** 1 Department of Clinical, Internal Medicine, Cardiovascular and Anesthesiological Sciences, “Sapienza” University of Rome, Viale del Policlinico 155, 00161 Rome, Italy; 2 Department of Medical Sciences, Azienda Ospedaliera Universitaria Senese, 53100 Siena, Italy; merlotti4@unisi.it

The history of vitamin D begins more than 100 years ago, with the initial documentation of rickets in industrialized cities of England. The disease associated with vitamin D deficiency was more noticeable in winter, having a lower incidence in summer. Further research led to the recognition of the importance of ultraviolet light. Finally, the entire chain of metabolites from ergocalciferol and cholecalciferol to the active 1,25(OH)2D was discovered and synthesized so that they could be used for pharmacological treatment [1,2,3,4]. However, in spite of this long scientific history, controversies still persist on numerous aspects; assay standardization, extra-skeletal effects, the role of precursors and metabolites of 25(OH)2D and therapeutic regimens are just a few [5].

This Special Issue on “The effect of vitamin D on metabolic bone disease and chronic diseases” attempts to address some of these controversies.

Recently, there has been a longstanding and unresolved debate regarding the role of vitamin D in influencing the prevalence and prognosis of COVID-19 patients [6,7,8,9,10,11,12]. Controversies also relate to the type of vitamin D to be used, including cholecalciferol, calcidiol and calcitriol in both prevention and treatment [6]. The manuscript of Mingiano et al. [13] evaluated the possible correlation between 25-OH vitamin D (25OHD) values and different conditions in hospitalized subjects with COVID-19 infection: mortality, prognosis, invasive and non-invasive mechanical ventilation and orotracheal intubation. The authors also analyzed the possible positive effect of calcifediol supplementation on COVID-19 severity and prognosis. They showed a positive correlation between circulating 25OHD levels and the partial pressure of oxygen and the FiO_2_ (PaO_2_/FiO_2_) ratio (r = 0.17; *p* < 0.05). Moreover, 25OHD levels were markedly reduced in patients who underwent non-invasive ventilation and orotracheal intubation. The length of hospitalization was greater in patients with severe 25OHD deficiency (<10 ng/mL), with significant difference also in mortality rate in relation to vitamin D status. The supplementation with calcifediol seemed to be effective in reducing the length of hospitalization and prognosis of COVID-19 patients.

The role of vitamin D in modulating cardiovascular risk has not been definitively settled. A number of studies suggest a possible association between vitamin D status and both cardiovascular risk and diseases such as hypertension, diabetes, obesity, coronary artery calcification, stroke and heart disease. In this context, both molecular and cellular pathophysiological mechanisms have been hypothesized including stress oxidative and inflammatory pathways [14]. However, there are inconsistent results considering the more recent megatrials [15,16,17,18,19]. Danese and coworkers [20] addressed this issue, investigating in 1240 blood donors (F/M ratio 1/3.2, mean age 41.9 years) the mutual interplay between bone, glucose and lipid metabolism in a wide cohort of community-based subjects. They found that vitamin D, parathyroid hormone, glucose and lipid metabolism are mutually influenced. Hypovitaminosis D predisposes toward worsening lipid profiles through the actions of parathyroid hormone, while serum hormone levels per se associate with higher glucose and LDL-cholesterol levels.

There is a universal need to reach vitamin D sufficiency in order to avoid the deleterious effects related to insufficiency or deficiency depending on the threshold chosen. Vitamin D3 can be obtained from the diet and by endogenous synthesis in the skin via the action of UVB radiation (290–315 nm), which converts 7-dehydrocholesterol to vitamin D3. Vitamin D2 is obtained only from the diet; it is naturally present in fungi (that is, wild mushrooms or UVB-treated cultivated mushrooms, yeasts). There are relatively few dietary sources of vitamin D3, the richest being oily fish and egg yolks. Other sources include meat/meat products and fortified foods, such as fat spreads, some breakfast cereals, some dairy products (especially yogurts) and vitamin D-fortified dairy alternatives. In this context, it is therefore fundamental to know the amount of vitamin D intake (that is, present in foods) to inform public health policies [21,22,23]. Nuti et al. [24] presented the results of a survey aimed to validate a specific frequency food questionnaire created to rapidly evaluate dietary vitamin D intake in Italian people. The data derived from this type of questionnaires were compared with the results derived in the same population sample from a 14-day frequency food diary. Both approaches demonstrated a remarkably low vitamin D intake, confirming that the vitamin D intake is very low in Italy and may contribute to hypovitaminosis.

Osteomalacia is the ominous consequence of long-standing vitamin D deficiency; it is an underdiagnosed disease. An exact or even an approximate prevalence of osteomalacia caused by vitamin D deficiency in the world is difficult to define because the condition is asymptomatic in most cases, especially in the elderly, or remains underrecognized in many cases [25]. There is a need for simple biochemical tests to assign the diagnosis without using invasive approaches. Al-Daghri and coworkers [26] attempted to make a diagnosis of biochemical osteomalacia combining measurement of four serum markers of hypomineralization, namely low 25 hydroxyvitamin D (25OHD < 30 nmol/L), high alkaline phosphatase, low calcium and/or inorganic phosphorous. They found that the overall prevalence of biochemical osteomalacia was 10.0% and was significantly higher in girls than boys. If further studies with the referent gold standard of histological evaluation confirm these results, there is no doubt that this biochemical approach can represent an easy-to-use tool to diagnose the disease.

Finally, the position statement of the Italian Society for Osteoporosis, Mineral Metabolism and Bone Diseases (SIOMMMS) [27] extensively addressed a number of issues (such as the definition of the vitamin D status; the opportunity of performing the biochemical assessment of serum 25(OH)D levels in the general population and in subjects at risk of hypovitaminosis D; how and whether to supplement vitamin D in subjects with hypovitaminosis D or candidates for pharmacological treatment with bone active agents; the safety of vitamin D). This represents a useful guidance for physicians in their daily clinical practice. Indeed, the Grading of Recommendations, Assessment, Development, and Evaluation (GRADE) system has been adopted. According to GRADE, evidence was revised based on five dimensions (risk of bias, imprecision, inconsistency, indirectness, publication bias) and categorized into four quality levels (high, moderate, low, or very low), while recommendations were classified as strong (“recommendations”) or weak (“suggestions”) on the basis of the quality of supporting evidence and level of agreement between the panel members.

In conclusion, these papers add important information to the ongoing debate surrounding vitamin D. However, independently of the different points of view, the most important message coming from this Special Issue on the effect of vitamin D on metabolic bone disease and chronic diseases resides in the importance of recognising vitamin D insufficiency and deficiency and in the unquestionable need of treating patients with these two conditions.
